# HIBLUP: an integration of statistical models on the BLUP framework for efficient genetic evaluation using big genomic data

**DOI:** 10.1093/nar/gkad074

**Published:** 2023-02-22

**Authors:** Lilin Yin, Haohao Zhang, Zhenshuang Tang, Dong Yin, Yuhua Fu, Xiaohui Yuan, Xinyun Li, Xiaolei Liu, Shuhong Zhao

**Affiliations:** Key Laboratory of Agricultural Animal Genetics, Breeding and Reproduction, Ministry of Education & College of Animal Science and Technology, Huazhong Agricultural University, Wuhan 430070, PR China; Frontiers Science Center for Animal Breeding and Sustainable Production, Wuhan 430070, PR China; School of Computer Science and Technology, Wuhan University of Technology, Wuhan 430070, PR China; Key Laboratory of Agricultural Animal Genetics, Breeding and Reproduction, Ministry of Education & College of Animal Science and Technology, Huazhong Agricultural University, Wuhan 430070, PR China; Key Laboratory of Agricultural Animal Genetics, Breeding and Reproduction, Ministry of Education & College of Animal Science and Technology, Huazhong Agricultural University, Wuhan 430070, PR China; Key Laboratory of Agricultural Animal Genetics, Breeding and Reproduction, Ministry of Education & College of Animal Science and Technology, Huazhong Agricultural University, Wuhan 430070, PR China; Frontiers Science Center for Animal Breeding and Sustainable Production, Wuhan 430070, PR China; School of Computer Science and Technology, Wuhan University of Technology, Wuhan 430070, PR China; Key Laboratory of Agricultural Animal Genetics, Breeding and Reproduction, Ministry of Education & College of Animal Science and Technology, Huazhong Agricultural University, Wuhan 430070, PR China; Frontiers Science Center for Animal Breeding and Sustainable Production, Wuhan 430070, PR China; Key Laboratory of Agricultural Animal Genetics, Breeding and Reproduction, Ministry of Education & College of Animal Science and Technology, Huazhong Agricultural University, Wuhan 430070, PR China; Frontiers Science Center for Animal Breeding and Sustainable Production, Wuhan 430070, PR China; Hubei Hongshan Laboratory, Wuhan 430070, PR China; Key Laboratory of Agricultural Animal Genetics, Breeding and Reproduction, Ministry of Education & College of Animal Science and Technology, Huazhong Agricultural University, Wuhan 430070, PR China; Frontiers Science Center for Animal Breeding and Sustainable Production, Wuhan 430070, PR China; Hubei Hongshan Laboratory, Wuhan 430070, PR China

## Abstract

Human diseases and agricultural traits can be predicted by modeling a genetic random polygenic effect in linear mixed models. To estimate variance components and predict random effects of the model efficiently with limited computational resources has always been of primary concern, especially when it involves increasing the genotype data scale in the current genomic era. Here, we thoroughly reviewed the development history of statistical algorithms used in genetic evaluation and theoretically compared their computational complexity and applicability for different data scenarios. Most importantly, we presented a computationally efficient, functionally enriched, multi-platform and user-friendly software package named ‘HIBLUP’ to address the challenges that are faced currently using big genomic data. Powered by advanced algorithms, elaborate design and efficient programming, HIBLUP computed fastest while using the lowest memory in analyses, and the greater the number of individuals that are genotyped, the greater the computational benefits from HIBLUP. We also demonstrated that HIBLUP is the only tool which can accomplish the analyses for a UK Biobank-scale dataset within 1 h using the proposed efficient ‘HE + PCG’ strategy. It is foreseeable that HIBLUP will facilitate genetic research for human, plants and animals. The HIBLUP software and user manual can be accessed freely at https://www.hiblup.com.

## Graphical Abstract

**Graphical Abstract:**
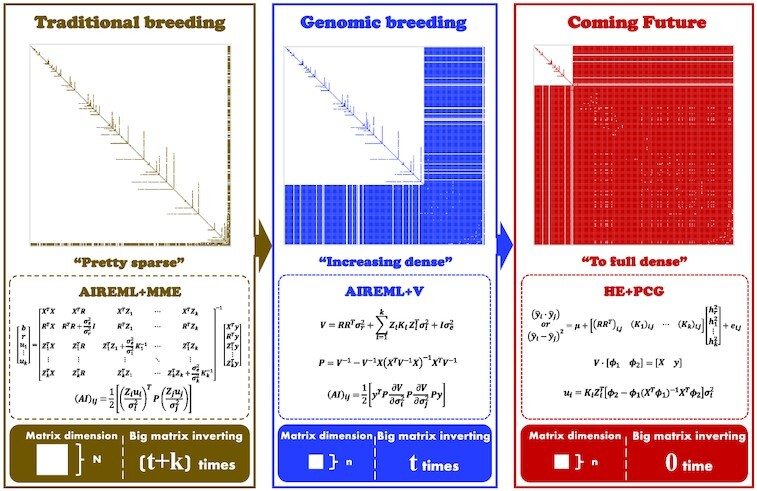
Challenges in genetic evaluation and corresponding solutions in HIBLUP. In the genomic era, existing genetic evaluation algorithms require the inverse computing on increasingly dense matrices. HIBLUP, implementing the “HE+PCG” strategy, can avoid the inverse computing of any big matrices.

## INTRODUCTION

As the most direct and effective measure to improve the individual performance on agricultural traits and to maximize economic benefits for breeding, genetic evaluation has experienced several major revolutions during the last half-century. Before the theory of genetics was proposed, breeders simply selected outstanding candidates based on their empirical experience by observing phenotypes. However, the phenotypic records of traits were the results of the combined influence of genetic and environmental factors; therefore, genetic progress for breeding was extremely slow due to the unquantified environmental contributions to phenotypic observations. In the mid-20th century, the BLUP (best linear unbiased prediction) model (1), which estimated the breeding values of individuals using the information from phenotypic observations, environmental records and a genetic relationship matrix that was derived from pedigree, was proposed and adopted in plant and livestock breeding, and it has resulted in great achievements in genetic improvements for agricultural economic traits. The great success of traditional pedigree-based genetic evaluation was largely attributed to state-of-the-art algorithms and efficient software implementations that used sparse technologies to estimate variance components and to solve mixed model equations (MMEs) for very large models (2,3).

With the development of sequencing technology, high-density genetic markers across the entire genome could be obtained, and genomic prediction, which was also known as genomic selection, was proposed subsequently (4). The utilization of genotypic information reduces Mendelian sampling error effectively, and more ideal models with flexible assumptions on the unknown genetic architecture of traits were developed (5–8). Therefore, the predictive accuracy of genomic prediction far outstripped traditional pedigree-based genetic evaluation, which led to genomic selection being promoted and widely applied in plant and livestock breeding (9). In the meantime, the number of genotyped individuals was increasing rapidly for some species. As an example, the number of genotyped Holstein cattle in the USA has been up to a scale of several millions (https://queries.uscdcb.com/Genotype/cur_freq.html). Using these large-scale datasets becomes more problematic for genetic evaluation, especially when the objective is to estimate variance components. This is because the algorithm implemented in most software tools [e.g. DMU (10), BLUPF90 (11) and ASReml (12)] requires several rounds of inversion of an increasingly dense coefficient matrix of MMEs; the benefits from traditional sparse technologies are limited and have encountered a bottleneck. Nowadays, much effort has been placed on developing faster and computationally feasible strategies to improve the ability to handle a larger number of genotyped individuals for MME-based algorithms (13,14). However, the benefits from the proposed strategies are still limited for the estimation of variance components, and the fatal defect of computing the inverse of genetic relationship matrices cannot be avoided when constructing an MME. Therefore, the increasing availability of genomic data requires the development of new software integrated with advanced algorithms to improve the computational efficiency of genetic evaluation.

The first purpose of this study was to review the development history of statistical algorithms used in genetic evaluation and to compare their computational complexity and applicability for different scenarios theoretically. The second purpose was to introduce a new computationally efficient and user-friendly software named ‘HIBLUP’, which has been enriched with almost all of the functionalities that are involved in genomic selection and genomic mating. Most notably, if the (co)variance components of traits are known, HIBLUP can obtain all the random effects quickly, which include breeding values, without the need to compute the inverse of any big matrices directly. Moreover, we compared it with other software tools or packages that are used widely regarding the computational efficiency and memory cost to demonstrate its huge application potential in genetic evaluation using big genomic data.

## MATERIALS AND METHODS

We consider the single trait linear mixed model


(1) $$\begin{equation*}y\; = \;Xb + Rr + \mathop \sum \limits_{i\; = \;1}^k {Z_i}{u_i} + e\end{equation*}$$

where $y$ is the vector of $n$ phenotypic records of the trait of interest, $X$ is the $n$ by ${n_b}$ design matrix for fixed effects and covariates, $b$ is the vector of corresponding effects, $R$ is the $n$ by ${n_r}$ design matrix for environmental random effects, $r$ is the vector of estimated effects that are assumed to be normally distributed on $N( {0,I\sigma _r^2} )$, $k$ is the total number of genetic random effects, ${Z_i}$ is the $n$ by ${n_i}$ design matrix for the ${i_{th}}$ genetic random effect and ${u_i}$ is the vector of its corresponding genetic effects with a length of ${n_i}$, which follows the multivariate normality of $N( {0,{K_i}\sigma _i^2} )$, ${K_i}$ can be the additive, dominant or other genetic relationship matrix that is derived from pedigree or genomic information, and the last term, $e$, is the vector of model residuals with a zero mean and a variance of $\sigma _e^2$.

The corresponding full rank MME for model (1) can be expressed as the following formula (1):


(2) $$\begin{equation*}\left[ {\begin{array}{@{}*{5}{c}@{}} {{X^T}X}& \quad {{X^T}R}& \quad {{X^T}{Z_1}}& \quad \cdots &\quad {{X^T}{Z_k}}\\ {{R^T}X}&\quad {{R^T}R + \frac{{\sigma _e^2}}{{\sigma _r^2}}I}&\quad {{R^T}{Z_1}}& \quad \cdots & \quad {{R^T}{Z_k}}\\ {Z_1^TX}& \quad {Z_1^TR}& \quad {Z_1^T{Z_1} + \frac{{\sigma _e^2}}{{\sigma _1^2}}K_1^{ - 1}}& \quad \cdots & \quad {Z_1^T{Z_k}}\\ \vdots & \quad \vdots & \quad \vdots & \quad \ddots & \quad \vdots \\ {Z_k^TX}& \quad {Z_k^TR}& \quad {Z_k^T{Z_1}}& \quad \cdots & \quad {Z_k^T{Z_k} + \frac{{\sigma _e^2}}{{\sigma _k^2}}K_k^{ - 1}} \end{array}} \right]\nonumber\\ \quad \left[ {\begin{array}{@{}*{1}{c}@{}} b\\ r\\ {{u_1}}\\ \vdots \\ {{u_k}} \end{array}} \right] = \left[ {\begin{array}{@{}*{1}{c}@{}} {{X^T}y}\\ {{R^T}y}\\ {Z_1^Ty}\\ \vdots \\ {Z_k^Ty} \end{array}} \right]\;\end{equation*}$$

This can be simplified to the form $C \cdot s\; = \;F$, where $C$ is generally known as the coefficient matrix. As shown in the above equation, the fixed and random effects that are located at the second term of the left-hand side of the MME can be obtained by multiplying the inverse matrix of $C$ by the right-hand side of the MME.

### Estimation of variance components

#### The MME-based indirect algorithm

To obtain the solution for Equation (2), the first step is to estimate the unknown variance components for all random effects. The restricted maximum likelihood (REML) is the method of choice to estimate model parameters (15). The aim of REML estimation is to find the parameters that maximize the logarithm of the restricted likelihood in the following form (16):


(3) $$\begin{eqnarray*}{\bf{ln}}L & = & - \frac{1}{2}\left[ {{\bf{ln}}\left| C \right| + {n_r}{\bf{ln}}\sigma _r^2 + \mathop \sum \limits_{i\; = \;1}^k \left( {n{\bf{ln}}\sigma _i^2 + {\bf{ln}}\left| {{K_i}} \right|} \right) + \left( {n - {n_b}} \right){\bf{ln}}\sigma _e^2 + {y^T}Py} \right]\nonumber\\ \end{eqnarray*}$$

where $\bf{ln}$ is a natural log, $P$ is the projection matrix, and | | refers to the determinant of the associated matrices. In the earlier stage, the derivative-free (DF) algorithm (17), which did not involve derivatives but used Gaussian elimination instead of computing ${C^{ - 1}}$ to obtain the key elements (${\bf{ln}}| C |$ and ${y^T}Py$) in Equation (3), was used widely in breeding evaluation due to its high computational efficiency. However, it was slow to converge, and it yielded unreliable results, especially for multiple traits or multiple random effects analyses (18). The expectation–maximization (EM) REML, which used the first-order derivatives of Equation (3), generated reliable results, but it took a long time to reach convergence. Nowadays, the average information (AI) REML, which requires first derivatives of the likelihood, but replaces second derivatives with the average of the observed and expected information, is a primary choice of methods to estimate variance components. As described by Johnson *et al.* (3), the equation for the iterative process of AIREML is


(4) $$\begin{equation*}{\theta ^{\left[ {t + 1} \right]}} = {\theta ^{\left[ t \right]}}\; - {(A{I^{\left[ t \right]}})^{ - 1}}\frac{{\partial L}}{{\partial \theta }}|{\theta ^{\left[ t \right]}}\end{equation*}$$

where $\theta \; = {[ {\sigma _r^2,\sigma _i^2,\sigma _e^2} ]^T}\;$, and ${\theta ^{[ {t + 1} ]}}$ and ${\theta ^{[ t ]}}$ are the vectors of variance components for current updates and previous estimations, respectively. The $AI$ matrix can be constructed by the following equation:


(5) $$\begin{equation*}\;{\left( {AI} \right)_{ij}} = \frac{1}{2}\;\left[ {{{\left( {\frac{{{Z_i}{u_i}}}{{\sigma _i^2}}} \right)}^T}P\left( {\frac{{{Z_j}{u_j}}}{{\sigma _j^2}}} \right)} \right]\end{equation*}$$

where ${u_i}$ is a vector of the ${i_{th}}$ genetic random effect, which can be obtained by solving Equation (2) directly. Let ${b_j} = {Z_j}{u_j}\;/\sigma _j^2$, then $P{b_j}$ in Equation (5) can be considered as the residuals from repeatedly solving the MME pertaining to Equation (2) with $y$ replaced by ${b_j}$. The first derivatives of the log likelihood function ($\frac{{\partial L}}{{\partial \theta }}$) with respect to each variance component are as follows:


(6) $$\begin{equation*}\begin{array}{@{}l@{}} \frac{{\partial L}}{{\partial \sigma _r^2}} = \; - \frac{1}{2}\left[ {\frac{{{n_r}}}{{\sigma _r^2}} - \frac{{tr\left( {{C^{rr}}} \right)}}{{\sigma _r^4}} - {{\left( {\frac{e}{{\sigma _e^2}}} \right)}^T}\left( {\frac{{Rr}}{{\sigma _r^2}}} \right)} \right]\\ \frac{{\partial L}}{{\partial \sigma _i^2}} = \; - \frac{1}{2}\left[ {\frac{n}{{\sigma _i^2}} - \frac{{tr\left( {K_i^{ - 1}{C^{{u_i}{u_i}}}} \right)}}{{\sigma _i^4}} - {{\left( {\frac{e}{{\sigma _e^2}}} \right)}^T}\left( {\frac{{{Z_i}{u_i}}}{{\sigma _i^2}}} \right)} \right]\\ \frac{{\partial L}}{{\partial \sigma _e^2}} = \; - \frac{1}{2}\left\{ {\frac{{n - {n_b}}}{{\sigma _e^2}} - \frac{1}{{\sigma _e^2}}\left[ {{n_r} - \frac{{tr\left( {{C^{rr}}} \right)}}{{\sigma _r^2}}} \right] - \frac{1}{{\sigma _e^2}}\mathop \sum \limits_{i\; = \;1}^k \left[ {{n_i} - \frac{{tr\left( {K_i^{ - 1}{C^{{u_i}{u_i}}}} \right)}}{{\sigma _i^2}}} \right] - \frac{{{e^T}e}}{{\sigma _e^4}}} \right\} \end{array}\end{equation*}$$

where $tr$ is the trace of matrix, $e$ is the vector of calculated residuals of model (1) after obtaining the solution of Equation (2), and ${C^{rr}}$ and ${C^{{u_i}{u_i}}}$ are the partitions of the inverse of the coefficient matrix that corresponds to environmental and the ${i_{th}}$ genetic random effects, respectively.

As shown above, the core steps of MME-based AIREML are the calculation of the inverse of the genetic relationship matrix to construct $C$ and the iterative computation of its inverse (${C^{ - 1}}$), which consume most of the time in the whole estimation process. Before genomic breeding was proposed, traditional breeding utilized the tracked pedigree records for individual evaluation, and the inverse of the additive genetic relationship matrix (${A^{ - 1}}$) required in constructing $C$ was derived from pedigree directly within seconds for tens of thousands of individuals using the algorithm described by Henderson (19). For calculating the matrix ${C^{ - 1}}$, although $C$ was very sparse for this case, its inverse had been regarded as expensive to calculate for large models. Misztal and Perez-Enciso proposed an efficient strategy which calculated only those elements of the inverse that belong to the sparse pattern of the original matrix (20), and then the fast sparse solver package FSPAK was developed and released to the public (21), making the traditional breeding evaluation superefficient. FSPAK has been implemented in various software packages, such as DMU (10) and BLUPF90 (11).

With the development of sequencing technology, high-density markers across the entire genome can be obtained and used to construct the genomic relationship matrix ($G$) by various algorithms (22–24), then genomic prediction came into the world in 2001 (4), and it has been promoted widely and applied in plant and livestock breeding in the past decade. SSGBLUP (single-step genomic BLUP) (25,26), which can integrate the pedigree and genotypic information simultaneously to predict both genotyped and non-genotyped individuals, has now become the preferred model in livestock breeding programs. Meanwhile, the ${H^{ - 1}}$ used to construct MMEs in the SSGBLUP model has dense blocks at the lower right corner due to the utilization of genomic information (see Supplementary Notes). The sparsity of ${H^{ - 1}}$ depends on the proportion of genotyped individuals in a pedigree; if all the individuals in a pedigree were genotyped, the ${G^{ - 1}}$ required in ${H^{ - 1}}$ had the same dimensions as ${A^{ - 1}}$, then SSGBLUP was equivalent to GBLUP and thus ${H^{ - 1}}$ became the full dense matrix. The increasing density of ${H^{ - 1}}$ limited the efficiency of sparse matrix operations in solving MMEs. To address this issue, a modified version of FSPAK with the new name of YAMS (Yet Another MME Solver) (13,27) was developed, which combined the supernodal factorization (28) and a supernodal version of the Takahashi algorithm to calculate the inverse of the coefficient matrix (29,30). YAMS has the ability to identify dense blocks and to compute them efficiently.

However, calculating the inverse of the genomic relationship matrix to construct a coefficient matrix is becoming computationally challenging due to the increasing number of genotyped individuals. The cost of dense matrix operations with GRM using YAMS is quadratic for memory and cubic for operations, which limits the computations to ∼50 000 animals. Subsequently, the Algorithm for Proven and Young (APY) strategy was presented (14,31), which considers that the genotyped individuals could be divided arbitrarily into core ($c$) and non-core ($n$) groups; the inverse of GRM can be calculated only on the condition of the genotypic information of core animals (see Supplementary Notes). The APY does not require computing the inverse of full matrix $G$, but instead it is required to invert the GRM of core animals ($G_{cc}^{ - 1}$). Since the number of core animals is usually smaller than the number of all genotyped individuals, the APY strategy not only reduces the complexity of inverse computation significantly, but also helps to increase the sparsity of the coefficient matrix; thus, it should be beneficial for both computational efficiency and memory usage. Nevertheless, how to select the core animals and how large the number of core animals should be to obtain unbiased results are not that easy to determine for a non-expert or non-experienced beginner (32). On the other hand, recent research concluded that to ensure reliable estimation of variance components, it is required to consider a core size that corresponds to the number of the largest eigenvalues that explain ∼98% of the total variation in GRM. Therefore, the savings in computing time to estimate the variance components are far less than the expected performance that the APY strategy has shown in solving MMEs. This inefficiency is because the block implementation of APY is still not possible for estimating variance components (33).

Although the optimizations of the MME-based algorithm have been in progress for several decades, the inverse computations of relationship matrices and of the coefficient matrix required to estimate variance components still face big challenges with the continuously growing size of genotyped individuals in plant and livestock breeding.

#### The V-based direct algorithm

In our developed software HIBLUP, we implemented another version of AIREML that could be more competent in breeding evaluation on big genomic data. By assuming multivariate normality of the phenotypic records $y\sim N( {Xb,\;V} )$, the variance–covariance matrix $V$ can be expressed as the following equation for model (1):


(7) $$\begin{equation*}V\; = \;R{R^T}\sigma _r^2 + \mathop \sum \limits_{i\; = \;1}^k {Z_i}{K_i}Z_i^T\sigma _i^2 + I\sigma _e^2\end{equation*}$$

The corresponding logarithm of the restricted likelihood, which is different from Equation (3), can be obtained using the following equation (34):


(8) $$\begin{equation*}{\bf{ln}}L\; = \; - \frac{1}{2}\left( {{\bf{ln}}\left| V \right| + {\bf{ln}}\left| {{X^T}{V^{ - 1}}X} \right| + {y^T}Py} \right)\end{equation*}$$

where $P$ is the projection matrix, which is defined as


(9) $$\begin{equation*}P\; = {V^{ - 1}}\; - {V^{ - 1}}X{\left( {{X^T}{V^{ - 1}}X} \right)^{ - 1}}{X^T}{V^{ - 1}}\end{equation*}$$

The iterative process for V-based AIREML is the same as Equation (4), but the construction of the AI matrix and the first derivatives of the log likelihood function differ from MME-based algorithms. The AI matrix could be written as follows (3):


(10) $$\begin{equation*}{\left( {AI} \right)_{ij}} = \frac{1}{2}\;\left[ {{y^T}P\frac{{\partial V}}{{\partial \sigma _i^2}}P\frac{{\partial V}}{{\partial \sigma _j^2}}Py} \right]\end{equation*}$$

and the first derivatives of the log likelihood function with respect to each variance component are


(11) $$\begin{equation*}\frac{{\partial L}}{{\partial \sigma _i^2}} = \; - \frac{1}{2}\left[ {tr\left( {P\frac{{\partial V}}{{\partial \sigma _i^2}}} \right) - {y^T}P\frac{{\partial V}}{{\partial \sigma _i^2}}Py} \right]\end{equation*}$$

From the above descriptions, the most time-consuming part for V-based AIREML is to calculate the inverse of the $V$ matrix in Equation (9); the other matrix operations can be computed efficiently. To speed up the efficiency of the V-based algorithm, an integration of eigen decomposition technology (35), sparsification processing on GRM using an empirical threshold and a log likelihood scores-based grid search (36) could be applied to the model with one genetic random effect. Supplementary Figure S1 illustrates the differences on estimation of variance components between the MME-based and V-based strategies. Compared with MME-based AIREML, V-based AIREML has the following advantages. (i) The dimension of the $V$ matrix only depends on the number of phenotypic records; it remains unchanged with the increasing number of random effects, while the dimension of the MME appears to have linear growth with the number of random effects and the number of levels in each random effect (generally the number of individuals in the pedigree). (ii) Only the partition of the relationship matrix among phenotypic individuals and the partition between phenotypic and non-phenotypic individuals are required; the partition of the relationship matrix among non-phenotypic individuals is not involved in operations and, therefore, is not necessary to be loaded into memory for the V-based algorithm. However, for the MME-based algorithm, all the elements in the relationship matrix participate in computing the inverse matrix. (iii) The $V$ matrix is the summation of (co-) variance matrices of all components, which is guaranteed to be positive definite for computing the inverse; moreover, we achieved Cholesky, lower–upper (LU) decomposition, and singular value decomposition (SVD) in HIBLUP to obtain the inverse or generalized inverse of the $V$ matrix. (iv) The V-based AIREML only involves the inverse calculation of the $V$ matrix, thus there is no need to compute the inverse of any relationship matrix, but the MME-based algorithm needs to compute the inverse for the coefficient matrix and for all genetic relationship matrices. (v) A practical application benefit of the V-based algorithm is that both pedigree-based and genotype-based relationship matrices can be updated on the basis of the prior calculated one when additional individuals are included in a new breeding task, which avoids repeat computations of the elements in the relationship matrix for the same individuals; however, it is nearly impossible to implement on an inverse matrix for the MME-based algorithm. (vi) The computational complexity and memory usage of V-based AIREML is mainly dependent on the number of phenotypic records, but it is dependent on the number of genotyped individuals for the MME-based AIREML. The only disadvantage of the V-based algorithm is that sparse matrix operations may no longer be applicable, especially for a model with a number of random effects; however, this is not significant because the matrices involved in the computation of genomic breeding are dense for the majority of cases.

#### The HE regression algorithm

In addition to REML analysis, a simple and approximate algorithm named HE (Haseman–Elston) regression, which estimates heritability directly by regressing pairwise similarities of phenotypic observations on pairwise similarities of genotypes, is an alternative (37). We have incorporated HE regression in HIBLUP and extended it to accommodate single trait and multiple traits models with multiple environmental and genetic random effects. The mathematical formula for the HE regression for model (1) is as follows:


(12) $$\begin{equation*}\begin{array}{@{}*{1}{c}@{}} {\left( {{{\hat y}_i} \cdot {{\hat y}_j}} \right)}\\ {{\rm{or}}}\\ {{{\left( {{{\hat y}_i} - {{\hat y}_j}} \right)}^2}} \end{array} = \;\mu + \left[ {\begin{array}{*{20}{c}} {{{\left( {R{R^T}} \right)}_{i,j}}}&{{{\left( {{K_1}} \right)}_{i,j}}}& \cdots &{{{\left( {{K_k}} \right)}_{i,j}}} \end{array}} \right]\left[ {\begin{array}{@{}*{1}{c}@{}} {h_r^2}\\ {h_1^2}\\ \vdots \\ {h_k^2} \end{array}} \right] + {e_{i,j}}\end{equation*}$$

where $i,j \in \{ {1, \cdots ,n} \}$, $\hat y$ is the adjusted phenotype of $y$ by fixed effects and covariates, and ${\hat y_i}$ and ${\hat y_j}$ are the adjusted phenotypic values of individuals $i$ and $j$ for the trait of interest, respectively. $\mu$ is the regression intercept, ${( K )_{i,j}}$ represents the element at row $i$ and column $j$ of the genetic relationship matrix $K$, ${h^2}$ is the estimated heritability of random effects and $e$ is a vector of residuals. The regression coefficients $\beta \; = \;c(h_r^2,h_1^2, \cdots ,h_k^2)$ in Equation (12) can be obtained easily by ordinary least squares (OLS) regression, then the variances of all random effects can be computed by ${\sigma ^2} = \;var( {\hat y} ) \cdot \beta$. For a two-trait model, replace the left-hand side of Equation (12) by $({\hat y_{1,i}} \cdot {\hat y_{2,j}})$ or ${( {{{\hat y}_{1,i}} - {{\hat y}_{2,j}}} )^2}$, and the covariance between two traits can be calculated by ${\sigma ^2} = \;cov( {{{\hat y}_1},{{\hat y}_2}} ) \cdot \beta$, then the genetic correlation can be calculated using the estimated variances and covariances. HE regression can be accomplished on-the-fly as it significantly reduces the computational burden with fewer matrix operations, no iterative processes and no involvement of computing the inverse of any big matrices such as a relationship matrix. Moreover, the memory cost can be decreased by several orders of magnitude, which makes it possible to estimate variance components for large datasets with limited computational resources.

It should be noted that previous studies indicated that the stability and power of HE regression varied with different genetic architectures of traits (38). In HIBLUP software, we provided an option named ‘HI’ for estimating variance components, which initializes the estimated outcomes of HE regression as the start values of AIREML. This can potentially help AIREML run in the correct iterative direction to overcome the ‘overshoot’ problem and help to converge in less time. To achieve reliable computing in estimation of variance components, a combination of different algorithms could be helpful (39). In HIBLUP, a series of algorithms that include AI, EM, AIEM, EMAI, HE and HI have been incorporated; users can switch easily to one of them and change the maximum iterative number for models with various complexities.

### Solving mixed-model equations

In practical breeding evaluations, estimating variance components does not need to run frequently. Estimation is generally updated quarterly or annually, and the most common computation is to solve the MME to obtain the estimated breeding values (EBVs) for all individuals with prior calculated variance components. Supplementary Figure S1 illustrates the detailed computing processes of the MME-based and V-based strategies.

For the MME-based algorithm, all random effects including EBVs can be obtained easily from Equation (2), and the simplified formula is


$$\begin{equation*}C \cdot s\; = \;F\end{equation*}$$

The matrix $C$ is most likely to be sparse if a large proportion of individuals in the pedigree are not genotyped, the iterative solvers [e.g. pre-conditioned conjugate gradient (PCG) and successive over-relaxation (SOR)] are preferred because there is no need to compute the direct inverse of the $C$ matrix. However, the operation of inverting the relationship matrix to construct the $C$ matrix in Equation (2) cannot be avoided.

For the V-based algorithm, EBVs can be obtained by the following equation:


$$\begin{equation*}{\rm{\;}}{u_i} = {K_i}\;Z_i^TPy\sigma _i^2\end{equation*}$$

The component $Py$ is the only term that needs to compute, as shown in Equation (9), and it can be written as


$$\begin{equation*}Py\; = {V^{ - 1}}\;y - {V^{ - 1}}X{\left( {{X^T}{V^{ - 1}}X} \right)^{ - 1}}{X^T}{V^{ - 1}}y\end{equation*}$$

Computing ${V^{ - 1}}$ directly is time expensive for a large matrix. Here in HIBLUP, we implemented a fast linear solver system to obtain the solution of ${V^{ - 1}}y$ and ${V^{ - 1}}X$ as follows:


$$\begin{equation*}V \cdot \;\left[ {\begin{array}{*{20}{c}} {{\phi _1}}&{{\phi _2}} \end{array}} \right] = \left[ {\begin{array}{*{20}{c}} X&y \end{array}} \right]\;\end{equation*}$$

The $V$ matrix is no longer sparse in the majority of cases for genomic breeding evaluation and the dimension of $X$ is far smaller than that of the $V$ matrix (${n_b} \ll n$); we implemented Cholesky and LU factorization-based linear solver for fast computing, and PCG is also included in HIBLUP as an alternative for very large data. Now, $Py$ can be calculated efficiently by using the following equation:


$$\begin{equation*}Py\; = {\phi _2}\; - {\phi _1}{\left( {{X^T}{\phi _1}} \right)^{ - 1}}{X^T}{\phi _2}\end{equation*}$$

Note that computing the inverse of ${X^T}{\phi _1}$ could be accomplished on-the-fly. Compared with the MME-based algorithm, the V-based algorithm can successfully avoid computing the inverse of any large matrix directly, and the dimension of the $V$ matrix is generally smaller than the dimension of MME, especially for a model with a large number of random effects. The dimension of the $V$ matrix remains unchanged with the number of random effects, while the dimension of MME appears to have linear growth, which results in an unpredictable amount of time spent and memory cost for analysis. Moreover, with the increasing number of genotyped individuals, the matrices involved in genomic breeding evaluation tend to be more and more dense than traditional breeding. The V-based algorithm should be more beneficial computationally than the MME-based algorithm, which is considered to be competent in a sparse matrix operation.

### Software development and algorithm implementation

#### Fast parallel computing

HIBLUP was developed in C++, and dense and sparse matrix implementations are conducted by Armadillo (40,41). The operations for dense matrices and vectors, and matrix decomposition (e.g. Cholesky, LU and SVD) are performed by the Basic Linear Algebra Subprograms (BLAS) and the Linear Algebra Package (LAPACK). Various math libraries have been linked for different platforms to speed up the computing efficiency, such as the MKL (Math Kernel Library) on Intel x86, the Apple Accelerate on both Intel x86 and ARM-based ‘M’ series, the OpenBLAS on ARM and the KML (Kunpeng Math Library) on ARM-based Huawei Kunpeng (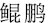). All the parallelizable loops (e.g. matrix filling) have been accelerated by OpenMP technology; some basic statistics, for example, genotype counting and allele frequency calculations, are accomplished by bit operation and a population count algorithm. We also designed and optimized the whole computational process to avoid repeat matrix operations across different functions. Here in HIBLUP, a look-up table (LUT) strategy was implemented for genotype numeric coding, and the accessed binary byte containing four individuals will be converted into an index to extract values from a prior designed array of 256 combinations (4^4, four possibilities for each of the four individuals). This strategy virtually digitalizes four individuals at a time by the trivial hash searching, rather than looping individuals one by one, and this improves efficiency significantly. We have developed a new function to compute a GRM in a very efficient manner with a very low cost of memory using block-wise operations.

#### Efficient memory management

In HIBLUP, we only store the lower triangle elements of a relationship matrix in a binary format file to save space in the local disk. To load the binary files of a relationship matrix and a Plink format genotype into memory efficiently, we use the memory-mapping technology with an integration of OpenMP, which can realize the fast access of any of the desired elements from a local disk rather than loading them all at one time with an expensive memory cost. We flexibly employ a mixture of float and double precision for matrix storage and operations in programming to reduce the memory usage for analysis.

#### Integration of abundant functionalities

In addition to implementing general genetic evaluation, including estimation of variance components, solving MMEs to obtain individual breeding values, HIBLUP has been enriched with many functionalities that are involved in genomic breeding. For example, these include statistical significance tests for fixed effects and covariates, fitting interactions among multiple effects (e.g. G × G, G × E and E × E), the construction of different relationship matrices, the derivation of inbreeding coefficients by using either or both pedigree and genotypic information, the calculation of genotype and allele frequency, the computation of single nucleotide polymorphism (SNP) effects, principal components analysis (PCA), genomic mating, and so on. We will continue to update HIBLUP with more features based on the users’ feedback, which should allow HIBLUP to always be fresh for general users and academic researchers.

#### Friendly user experience

HIBLUP is a command-line program that relies on text inputs to perform the analysis, and the names of parameters and options in HIBLUP are quite consistent with those widely used in software tools (e.g. Plink and GCTA) so as to satisfy the programming habits of users. Users can achieve simple linear models, linear mixed models, pedigree-based BLUPs, GBLUPs or SSGBLUPs, and specify fixed or environmental random effects with just a few commands. Moreover, we have released multiple versions of executable files for the platform of Linux, MacOS, Windows and particularly Kunpeng (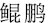), which ensures that HIBLUP has wider application scenarios.

## RESULTS

To evaluate the computational effectiveness of different versions of AIREML that are implemented in various software tools for estimating variance components in genomic breeding evaluation, we simulated the experimental population data using the R package Simer. A total of 1000 individuals that consisted of 500 males and 500 females with 100 000 SNPs were treated as the base population, that was the first generation. Nine generations of random mating without backcross were then simulated with considering a litter size of two (one male and one female). Finally, a historical population of 10 000 individuals with detailed pedigree and genotypic information was generated for the downstream evaluation. Although the simulation scheme was not fully consistent with practical breeding, the simulated data structure was sufficient to evaluate the computational efficiency of the algorithm implemented in different software tools. We only simulated 10 000 individuals because the publicly available version of BLUPF90 used for computational comparison has a limitation for non-commercial analysis. Since GCTA only supports two traits at a time for a multiple traits model, two correlated traits that were controlled by 600 randomly selected SNPs with effect size normally distributed at zero mean and a variance of one were simulated at a heritability of 0.1 and 0.3, respectively. The genetic correlation was set to 0.3. Additionally, two environmental random effects and two fixed effects were added to the final phenotypic records for each trait. The computational comparisons were tested on a Linux server, the detailed system information of which had been described in the notes of each table, and the time and memory costs were recorded by running the systematic function ‘*/usr/bin/time -v*’ with a tail of the prepared scripts of different software tools. We used the same convergent precision 1e-8 and set the maximum iteration number of 100 for all software tools, but we did not assign the start values, because how to automatically provide more appropriate start values to achieve faster iterative convergence for the traits with unknown genetic architecture is one of the key factors of computational efficiency of a software tool.

We first compared the computational efficiency for estimation of variance components for the most widely used parallelizable software tools or packages in the domain of genomic breeding evaluation by running the GBLUP model, which only requires genotypic information for prediction and is considered to be the most computationally expensive model in genetic evaluation. The software tools of choice were BLUPF90 (11), ASReml (12), GCTA (42), MTG2 (35), LDAK (43) and Sommer (44). Here, we did not include DMU (10) because the released version of DMU had no availability of parallel computing. All the selected software tools above were run in parallel automatically by the aid of packaged Intel MKL, except for the Sommer package; we ran Sommer in Microsoft R Open (MRO) (https://mran.microsoft.com/) to achieve parallel computing. When evaluating all the software tools or packages, we first computed the relationship matrix or its inverse, then used it directly for the downstream estimation of variance components and derivation of breeding values. When fitting a single trait model, two fixed effects and two environmental random effects were added in the analysis. Because parts of software tools (e.g. GCTA and LDAK) have no corresponding options to process environmental random effects, we prepared the relationship files manually in the required format by using HIBLUP. For a multiple traits model, we did not add any fixed effects and environmental random effects in the analyses because GCTA had no corresponding function; only one additive genetic random effect was fitted in the model for estimating genetic correlations. For BLUPF90, since it has no relevant function for fitting the GBLUP model, we ran the SSGBLUP model instead; the option ‘*use_yams*’ for YAMS was opened for a fast computing block dense matrix, but the APY strategy was turned off to ensure the same results from different software tools. For ASReml, we ran ASREML-SA instead of ASReml-R for parallel computing, and its required inverse of the relationship matrix was constructed by the R package ASRgenomics on the platform of MRO.

**Figure 1.**
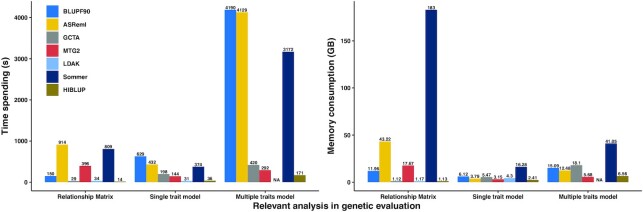
Comparisons of computational cost on time and memory of variance components estimation for the GBLUP model. The relationship matrix was constructed for all 10 000 genotypic individuals, and the computational costs were recorded for the construction of the inverse of the relationship matrix for the MME-based strategy (BLUPF90 and ASReml). ‘NA’ means the corresponding function is unavailable. The number of phenotypic records is 10 000 for both single trait and multiple traits models. All results were computed on a Red Hat Enterprise Linux server with 2.20 GHz Intel(R) Xeon(R) 32 CPUs E7-8880 v4 and 125 GB memory.

The running script and log information for different software tools have been provided in Supplementary File S1, and the compared results are shown in Figure 1 and Supplementary Table S1. The ‘*G*^−1^’ in the table represents those software tools that were compiled into the MME-based strategy, and the others implemented the V-based strategy. For GRM construction, HIBLUP computed the fastest, and it was accomplished within 15 s by using 32 threads for a population at a scale of 10 000 individuals and 100 000 SNPs. Because HIBLUP can also output the inverse of GRM directly, we also demonstrated the efficiency of HIBLUP for calculating the inverse matrix, it was amazing that HIBLUP only spent 3 s to compute the inverse of a matrix with a dimension of 10 000 × 10 000 while the memory cost remained unchanged (Supplementary Table S1). GCTA performed similarly to HIBLUP when constructing the GRM matrix; the Sommer package required a massive amount of computing time and memory, which was attributed to the unavailability of single floating-point operations and the lagging memory management and collection mechanism in R language. Roughly speaking, the computational time spent and memory cost to construct GRM and to compute its inverse for the software tools that implemented the MME-based algorithm were nearly 10 times greater than for HIBLUP.

For estimation of variance components, all these software tools generated nearly the same results as shown in Supplementary Table S2. BLUPF90 exhibited a negligible difference from the other tools—this was because BLUPF90 utilized the pedigree information in analysis—and HIBLUP had the smallest iterative number for either a single trait model or a multiple traits model. For a single trait model, both HIBLUP and LDAK finished within 30 s, but HIBLUP used the lowest memory for analysis, and BLUPF90 required the longest time. For the multiple traits model, the memory costs of MTG2 and HIBLUP were 2–7 times less than for other software tools, and HIBLUP was the most efficient computationally. We found that even though ASReml had a smaller iteration number (9) than BLUPF90 (15), ASReml still required a similar computational time to BLUPF90, which meant that ASReml needed much more time per iteration than BLUPF90. Because the GBLUP model only used genotypic information rather than pedigree records, there were no mathematical operations related to sparse matrices. Undoubtedly, from the results above, we concluded that the software which implemented the V-based algorithm was more computationally beneficial than that which relied on the MME-based indirect algorithm when the genetic evaluation merely involved genotypic information.

In practice, SSGBLUP has become the first choice in livestock breeding owing to its flexible integration of pedigree and genotypic information. The computational complexity of the SSGBLUP model depends mainly on the number of phenotypic records and the number of genotyped individuals in the pedigree. To systematically investigate the computational efficiency and the applicability of HIBLUP in a realistic evaluation of breeding, we designed a series of combinations of different numbers of phenotypic records and genotyped individuals to reflect the various complexities of genomic breeding data structure. Then, we employed it to evaluate the computational efficiency of HIBLUP with the software BLUPF90, which is used commonly to fit the SSGBLUP model in livestock genomic breeding. The genotyped individuals were selected in order from the last generation to the first generation at 625, 1250, 2500, 5000 and 10 000 out of 10 000 individuals in the pedigree; the individuals with phenotypic records were selected randomly from the 10 000 phenotypic records at the same levels, and the individuals with phenotypic records in the current level fully contained all the individuals from the previous level.

In the experiments, we did not fit any environmental random effects, but only included one genetic random effect in either single trait or multiple traits models with the consideration that too many random effects would cause convergent failure for limited phenotypic records (e.g. 625). Table 1 reports the compared results on estimation of variance components using various complexities of data structure. For relationship matrix construction, computing the H matrix by HIBLUP was always more efficient than deriving its inverse matrix by using BLUPF90, and the memory consumption of HIBLUP grew more slowly with an increasing number of genotyped individuals. For estimation of variance components, a quick view of Table 1 shows that HIBLUP was computationally faster than BLUPF90 for most scenarios, especially when the breeding data have few phenotypic records and a tremendous number of genotyped individuals that are needed to predict, which meets the early stage selection in practical breeding. If the number of phenotypic records was constant, the time spent and memory cost stayed nearly the same for HIBLUP when more individuals were genotyped, but increased with an increasing number of phenotypic records; it was just the opposite for BLUPF90. The performance for time and memory of HIBLUP was more stable across different structural datasets, which makes HIBLUP predictable with respect to the requirements of computational resources for any scale of data at hand. We also found that BLUPF90 tended to be more difficult to reach convergence with a decreased number of phenotypic individuals, especially for a multiple traits model. No matter how big or small the number of phenotypic records is, the MME-based strategy in BLUPF90 requires construction of an MME, the dimension of which is equal to the number of individuals in the pedigree. However, in the V-based strategy, the dimension of the *V* matrix is equal to the number of individuals with phenotypic records, which is far smaller than the number of individuals in a pedigree for the majority of cases. Due to the limited information provided by a smaller number of phenotypic records, a bigger matrix requires much more time for the iterative process to be converged for the AIREML or PCG algorithm, especially for an increasingly denser matrix. This is the reason why BLUPF90 has erratic performances across different numbers of phenotypic records and genotyped individuals.

**Table 1.** Comparisons of computational cost in time and memory of estimation of variance components for the SSGBLUP model

					**Number of phenotypic records**									
**Time (s)/memory (GB)**	***N***	**Software**	**Relationship matrix**		**Single trait model**					**Multiple traits model**				
					625	1250	2500	5000	10 000	625	1250	2500	5000	10 000
**Number of genotyped individuals**	625	BLUPF90	*H* ^−1^	8/0.67	38/0.13	22/0.27	109/0.21	77/0.14	43/0.12	1358/0.36	103/0.59	94/0.65	425/0.49	101/0.65
	1250			12/1.25	53/0.39	36/0.40	131/0.46	120/0.46	72/0.40	2226/0.91	330/0.69	206/0.58	1318/0.59	312/0.56
	2500			25/2.42	138/0.65	102/0.55	208/0.65	197/0.60	173/0.64	4371/1.33	233/1.61	240/1.60	1223/1.56	421/1.67
	5000			52/5.41	360/1.64	685/1.95	453/1.97	432/2.01	217/1.98	6512/4.24	495/4.23	494/4.28	2126/4.51	557/4.23
	10 000			150/11.96	787/6.10	1533/6.11	1468/6.14	1268/6.28	865/6.18	25 445/15.12	1939/15.14	5633/15.28	6088/15.11	4190/15.09
	625	HIBLUP	*H*	6/1.09	1/0.23	1/0.27	4/0.37	13/0.88	56/2.39	1/0.23	3/0.32	9/0.81	32/2.16	204/7.04
	1250			7/1.10	1/0.23	1/0.27	4/0.37	13/0.89	47/2.38	3/0.27	5/0.33	11/0.81	35/2.18	205/6.98
	2500			10/1.13	1/0.24	1/0.26	3/0.37	11/0.92	44/2.37	2/0.26	3/0.32	10/0.84	36/2.15	193/7.17
	5000			18/1.28	1/0.24	1/0.27	3/0.37	11/0.93	40/2.38	2/0.25	3/0.32	10/0.84	36/2.19	171/7.11
	10 000			58/1.94	1/0.24	1/0.26	3/0.38	11/1.09	49/2.51	2/0.25	3/0.32	9/0.82	45/2.29	213/7.49

Values in each cell represent ‘time (s)/memory (GB)’, ‘*H*’ is the relationship matrix derived from the pedigree (*n*= 10 000) and genotypic information (*N*), and the genotyped individuals are randomly selected from pedigree, ‘*H*^−1^’ is the inverse of ‘*H*’. The total number of predicted individuals is 10 000. All the experiments were computed on a Red Hat Enterprise Linux sever with 2.20 GHz Intel(R) Xeon(R) 32 CPUs E7-8880 v4 and 125 GB memory.

Finally, we compared the efficiency of solving an MME to obtain EBVs using the prior estimated variance components. Note that the default method for a linear solver in BLUPF90 was PCG, but it was Cholesky or LU decomposition in HIBLUP. From Table 2 we can see, when the product of the number of phenotypic records and the number of traits was within 5000, HIBLUP output the EBVs in just 1 s, and no matter how many individuals we genotyped, the required memory remained unchanged if the number of phenotypic individuals remained constant. However, for BLUPF90, the time spent was several hundred times longer than for HIBLUP, the memory cost increased significantly with the increasing number of genotyped individuals and the PCG iteration in BLUPF90 required more time to converge when there were only a few phenotypic records for a large number of genotyped individuals. However, when there were a tremendous number of phenotypic individuals but only a small proportion of individuals in the pedigree were genotyped, BLUPF90 demonstrated superior performance due to its advanced sparse technology. We also compared the computational performances of decomposition and PCG algorithms implemented in HIBLUP (Supplementary Table S3). The results showed that the decomposition and PCG algorithms had similar performances on the scale of simulated datasets regarding the time spent, and only small differences were found when the number of phenotypic records reached 10 000, but the decomposition algorithm required higher memory than PCG, which was attributed to the newly generated matrix to store the decomposition results. Although PCG has far less complexity than decomposition, it is an iterative algorithm which cannot be paralleled theoretically. Instead, the computational efficiency of matrix decomposition could be greatly improved by parallel computing. Therefore, the parallel-accelerated decomposition has faster computational performance than PCG on a small or medium population size for most users. However, for a very large population, the PCG algorithm should be the first choice.

**Table 2.** Comparisons of computational cost in time and memory of solving a mixed model equation for the SSGBLUP model

			**Number of phenotypic records**									
**Time (s)/memory (GB)**	***N***	**Software**	**Single trait model**					**Multiple traits model**				
			625	1250	2500	5000	10 000	625	1250	2500	5000	10 000
**Number of genotyped individuals**	625	BLUPF90	1/0.04	1/0.04	1/0.03	1/0.04	1/0.04	3/0.09	3/0.09	2/0.09	2/0.09	2/0.09
	1250		2/0.06	2/0.06	1/0.06	1/0.06	2/0.1	8/0.17	8/0.17	7/0.17	7/0.17	6/0.17
	2500		9/0.20	9/0.20	7/0.20	5/0.20	5/0.20	39/0.63	32/0.63	28/0.63	23/0.62	22/0.62
	5000		21/0.76	20/0.76	22/0.76	21/0.76	18/0.76	172/2.45	144/2.45	103/2.45	106/2.45	97/2.45
	10 000		97/3.01	111/3.01	81/3.01	63/3.01	54/3.01	724/9.76	613/9.76	483/9.76	370/9.76	327/9.76
	625	HIBLUP	1/0.21	1/0.24	1/0.28	1/0.63	2/1.41	1/0.21	1/0.24	1/0.54	2/1.23	9/3.68
	1250		1/0.21	1/0.24	1/0.28	1/0.63	2/1.41	1/0.21	1/0.24	1/0.54	2/1.22	9/3.67
	2500		1/0.21	1/0.24	1/0.28	1/0.63	2/1.41	1/0.21	1/0.24	1/0.54	2/1.24	9/3.67
	5000		1/0.21	1/0.24	1/0.28	1/0.63	2/1.43	1/0.21	1/0.23	1/0.54	2/1.25	9/3.66
	10 000		1/0.21	1/0.24	1/0.28	1/0.71	2/1.54	1/0.21	1/0.24	1/0.54	2/1.36	11/3.83

Values in each cell represent ‘time (s)/memory (GB)’. The genotyped individuals are randomly selected from the pedigree, and the total number of predicted individuals is 10 000. All the experiments were computed on a Red Hat Enterprise Linux sever with 2.20 GHz Intel(R) Xeon(R) 32 CPUs E7-8880 v4 and 125 GB memory.

Although the observed computational benefits from HIBLUP over other software tools had been outstanding for the one or two traits models with only 10 000 individuals, we further simulated a UK Biobank scale dataset consisting of 500 000 individuals and 1 million markers, then used it to evaluate the computational efficiency of HIBLUP and GCTA (which had the performance closest to that of HIBLUP in Figure 1 and Supplementary Table S1). The compared results showed that HIBLUP can accomplish the GRM construction within 21 h at a memory cost of 1 TB using 128 threads, while GCTA took more than twice the computational time (56 h) and memory (2.3 TB) compared with HIBLUP at the same level of threads (see Supplementary File S2). Moreover, the GRM construction in HIBLUP can be speeded up by assigning a local file listing a subset of SNPs that are filtered at a user-specific condition, such as clumping, pruning, and so on, and we also have provided an option ‘–step’ to enable the users to balance the memory usage and time spent according to the size of computational resources. We then tried to estimate variance components and to derive the genomic breeding values on this large dataset; HIBLUP can finish all the analyses in 1 h at a memory cost of 1.8 TB owing to the implemented efficient ‘HE + PCG’ strategy (Supplementary File S2), where ‘HE’ and ‘PCG’ are the methods used in estimation of variance components and genomic breeding value derivation, respectively. As GCTA can fit the HE regression but has no direct functionality of solving MMEs, we first ran HE regression to estimate the variance components, then assigned it as the start values of AIREML with just one iteration to derive the breeding values, which we call the ‘HE + Solver’ strategy. The results showed that GCTA spent 3 h to finish the HE regression analysis, and required a memory cost of 5.5 TB when estimating the breeding values. However, the program was still in computing after running for 100 h; we had to stop it because of the uncertain runtime. This meant that the ‘HE + PCG’ strategy in HIBLUP was at least 100 times faster than the ‘HE + Solver’ strategy in GCTA. Based on the results of Figure 1, it is safe to say that the other software tools would require more days or even several weeks by using the available strategies (e.g. AIREML + PCG, AIREML + Solver and AIREML + FSPAK/YAMS). As we discussed, this big computational advantage of HIBLUP was largely attributed to the fact that the ‘HE + PCG’ strategy does not require computation of the inverse of any big matrix in either variance component estimation or breeding value derivation. We therefore believe that the advantages on computational efficiency of HIBLUP over other software tools would be even much larger with an increasing number of traits and an increasing number of genotyped individuals in the model using more computational resources in the analysis. This makes HIBLUP a very promising tool in genetic evaluation using big genomic data.

## DISCUSSION

In the above sections, we theoretically described and compared the computational complexity between the MME-based indirect algorithm and the V-based direct algorithm in genetic evaluation. We concluded that HIBLUP was much more competent in genomic breeding evaluation than existing tools that implemented an MME-based strategy because of the efficient implementations of the V-based algorithm. The high efficiency of V-based algorithms is attributed to the parallelizable decomposition on dense matrices in estimation of variance of components and the non-requirement for directly inverting any relationship matrices in derivation of breeding values. However, it should be mentioned that if the evaluation only involves the pedigree (i.e. traditional breeding) or there is a very large pedigree in which only a very small proportion of individuals are genotyped, the software that implements the MME-based algorithm should be the first choice to handle this kind of data because the inverse of the relationship matrix is pretty sparse, and it can be obtained extremely quickly. On the contrary, the relationship matrix required for the V-based algorithm is generally dense in this situation, and factorization and inversion of a dense matrix could be much more time and memory expensive than on a very sparse matrix. However, on the one hand, if the pedigree-based relationship between the predicted generation and their ancestors beyond 3–5 generations has become very weak, the prediction accuracy of the predicted generation would not be improved much when the depth of pedigree records exceeds five generations (45,46), and evidence showed that the convergence rate of the model can be undesirable for large pedigrees; a pedigree depth of three generations was optimal for convergence (47). Instead, the prediction accuracy may be decreased because the continuous artificial selection results in a big genetic gap between two remote generations and, therefore, the scale of pedigree used in genetic evaluation would not be very large; on the other hand, with the application of genomic selection in practical breeding year after year, all the individuals in performance testing would be genotyped and, therefore, all the phenotypic individuals would have genotypic information. The genetic relationship between generations can be measured by genotypic information directly; the model that can utilize the big genotype data and phenotypic records effectively should be the ‘The Chosen One’ in future genetic evaluation. All in all, the scale of data currently used in genetic evaluation, including phenotype, genotype and pedigree, would be generally within 3–5 generations, and the proportion of genotyped individuals in the pedigree is increasingly larger, which is very friendly to the V-based algorithm. However, for the MME-based algorithm, the effectiveness of computational efficiency for sparse technologies on a coefficient matrix will be limited, the process of locating sparse elements may lower the computation efficiency, and the memory cost will be several times larger than the V-based algorithm as the coefficient matrix of the MME and its inverse are no longer sparse for this case.

Nowadays, general genetic evaluation only accounts for the additive genetic effect in a model because the additive genetic effect is the only one considered to be heritable across generations. However, non-additive genetic effects may have an important contribution to the total genetic variation of complex traits, especially for a hybrid or crossbred population. Published papers have indicated that the model that includes additive and non-additive genetic effects can be beneficial for both genomic prediction (48) and genomic mating (49). In addition, the high-density SNPs across the entire genome are the mainstream markers used to implement genomic prediction, because it is common sense that the causal mutations of a trait are most likely to be in linkage disequilibrium (LD) with at least one SNP. However, a single piece of information from SNPs cannot fully capture the genetic variation of a trait. Additionally integrating structure variants (SVs) of genomics, including deletions, insertions, duplications, inversions and translocations, into a model with multiple genetic random effects can improve the prediction accuracy (50,51). Moreover, because of the fast development of sequencing technology, the increasing size of other non-genomics data (e.g. transcriptomics, proteomics and metabolomics) can be accessed freely by the public, and these multi-omics data provide a bridge between organismal phenotypes and genomics, and could bring new insights to the understanding of genetic architecture of traits. Thus they can be utilized in fitting a model to potentially improve the prediction performance (52,53). Additionally, because different levels of data may provide shared genetic information, the covariance terms between these random effects can be substantial, so fitting interactions between genetic random effects in the model is necessary (54). In brief, multiple genetic effects, multiple types of variants, multiple omics data and multiple interactions within random effects will be considered in mixed models to improve prediction accuracy. None of these terms is sparse in computation; therefore, it would be a huge challenge, if not nearly impossible, to fit such a large number of genetic random effects in an MME-based algorithm, because the dimension of an MME appears to increase linearly with the number of environmental and genetic random effects, which results in unpredictable time and memory costs for analysis. However, for the V-based algorithm implemented in HIBLUP, the computational burden mainly depends on the dimension of the V matrix, which is equal to the number of phenotypic records; no matter how many random effects are fitted in the model, the dimension of the V matrix stays the same. Only a small fraction of time is required to reach an iterative convergence; thus, the computing efficiency for the model of multiple genetic random effects is quite consistent with that of a single genetic random effect, making HIBLUP more promising for genetic evaluation in the genomic era compared with existing software tools.

We conclude that the V-based algorithm is more computationally advantageous compared with the MME-based algorithm for genomic breeding; the highly efficient ‘HE + PCG’ strategy implemented in HIBLUP can avoid computing the inverse of any matrix, and no doubt it should be the most appropriate solution for genetic evaluation using large genomic data. However, the real problem is that all these kinds of algorithms require the construction of a relationship matrix or its inverse matrix. However, with the rapidly increasing number of individuals that are genotyped, the size of the relationship matrix exhibits square growth. Leaving aside the time used in computing, the memory needed for storing such a large matrix may be up to several terabytes. To address this issue, we could make a dense matrix more sparse manually; however, this may generate biased estimation of variance components for an MME-based algorithm (33) or only work for a model with just one genetic random effect for a V-based algorithm (36). Another strategy that may be more applicable is to transform the determinant of computational burden into the number of markers from the number of individuals. Because most species just use a chip array (e.g. 50–80k for pigs) for genomic breeding rather than sequencing data, the number of individuals is far larger than the number of markers in this situation. Therefore, it is feasible to regress the phenotypic records directly on all markers simultaneously using a multiple regression model (MRM) to obtain the joint marker effects, the EBVs and variance components rapidly. Except for MRM, summary statistics can also be adaptable; it first fits a single marker regression model to get the marginal effects and standard error of all markers (i.e. summary-level data), then uses the LD score regression to estimate the variance components (55) and fit the SBLUP (summary-level BLUP) model (56) to obtain the joint marker effects. The strategies discussed above can successfully eliminate constructing a relationship matrix or its inverse matrix that is used in genetic evaluation, but this should be explored further and investigated by using real breeding data, which would be our next focus of work on HIBLUP in the future.

In conclusion, to improve the efficiency of genetic evaluation in the genomic era, we presented a computationally efficient, functionally enriched and user-friendly software called ‘HIBLUP’. Powered by the new advanced algorithms, the elaborate computational design and the efficient software programming, HIBLUP computed the fastest, required the least memory for analysis and the computational benefits from HIBLUP over other software tend to be greater with an increasing number of genotyped individuals. Owing to the user-friendly software design, abundant functional modules, comprehensive logging information and clear reminder of events, HIBLUP is simple to start, easy to learn and flexible to use.

## DATA AVAILABILITY

The HIBLUP software and user manual can be accessed freely at https://www.hiblup.com.

The software packages and tools used were Simer, https://CRAN.R-project.org/package=simer; ASRgenomics, https://asreml.kb.vsni.co.uk/asrgenomics-download-success; ASReml, https://vsni.co.uk/software/asreml; BLUPF90, http://nce.ads.uga.edu/html/projects/programs/; GCTA, https://yanglab.westlake.edu.cn/software/gcta; LDAK, https://dougspeed.com/ldak/; MTG2, https://sites.google.com/site/honglee0707/mtg2; and Sommer, https://CRAN.R-project.org/package=sommer.

## Supplementary Material

gkad074_Supplemental_Files
