# Supplementary material for: HIBLUP: an integration of statistical models on the BLUP framework for efficient genetic evaluation using big genomic data
Source: Nucleic Acids Res. 2023 Feb 22;51(8):3501–12. doi: 10.1093/nar/gkad074 (PMC10164590; doi:10.1093/nar/gkad074)
Supplement: gkad074_Supplemental_Files [file gkad074_supplemental_files.zip › Supplementary Figure S1.docx]

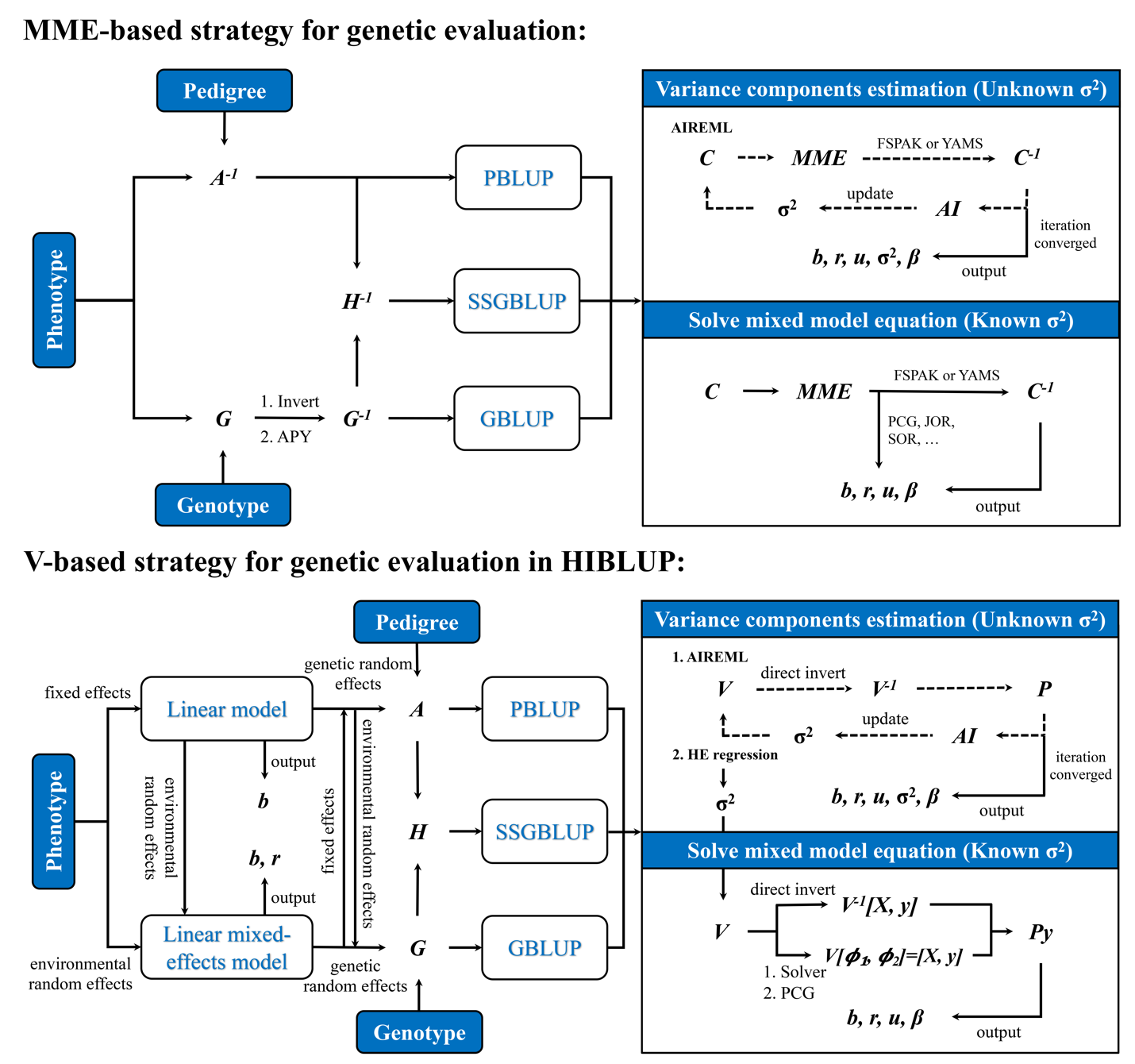


**Supplementary Figure S1. Illustration of the differences between MME-based strategy and V-based strategy for genetic evaluation.** The symbols ‘***A***’, ‘***G***’, ‘***H***’ are the relationship matrices derived from different files, and ‘***b***’, ‘***r***’, ‘***u***’, ‘***β***’, ‘***σ^2^***’, ‘***C***’, ‘***P***’, and ‘***AI***’ are the estimated coefficients of fixed effects, environmental random effects, genetic random effects, SNP effect size, variance components, coefficient matrix of MME, projection matrix, and average information matrix, respectively. All these mathematical symbols are completely consistent with that in the main text.
