# Supplementary material for: HIBLUP: an integration of statistical models on the BLUP framework for efficient genetic evaluation using big genomic data
Source: Nucleic Acids Res. 2023 Feb 22;51(8):3501–12. doi: 10.1093/nar/gkad074 (PMC10164590; doi:10.1093/nar/gkad074)
Supplement: gkad074_Supplemental_Files [file gkad074_supplemental_files.zip › Supplementary materials.docx]

**Supplementary materials to**

**HIBLUP: An integration of statistical models on the BLUP framework for efficient genetic evaluation using big genomic data**

Lilin Yin^a,b,1^, Haohao Zhang^c,1^, Zhenshuang Tang^a^, Dong Yin^a^, Yuhua Fu^a,b^, Xiaohui Yuan^c^, Xinyun Li^a,b,*^, Xiaolei Liu^a,b,d,*^, Shuhong Zhao^a,b,d,*^

*^a^ Key Laboratory of Agricultural Animal Genetics, Breeding and Reproduction, Ministry of Education & College of Animal Science and Technology, Huazhong Agricultural University, Wuhan 430070, PR China*

*^b^ Frontiers Science Center for Animal Breeding and Sustainable Production, Wuhan 430070, PR China*

*^c^ School of Computer Science and Technology, Wuhan University of Technology, Wuhan 430070, PR China*

*^d^ Hubei Hongshan Laboratory, Wuhan 430070, PR China*

*^1^ Equal contribution.*

*^*^ Correspondence: Shuhong Zhao (*[*shzhao@mail.hzau.edu.cn*](http://shzhao@mail.hzau.edu.cn)*), Xiaolei Liu (*[*xiaoleiliu@mail.hzau.edu.cn*](http://xiaoleiliu@mail.hzau.edu.cn)*), Xinyun Li (*[*xyli@mail.hzau.edu.cn*](http://xyli@mail.hzau.edu.cn)*).*

**Supplementary Notes**. The mathematically detailed descriptions for parts of methods in genetic evaluation.

**Supplementary Figure S1**. Illustration of the differences between MME-based strategy and V-based strategy for genetic evaluation.

**Supplementary Table S1**. Comparisons of computational cost on time and memory of variance components estimation for GBLUP model.

**Supplementary Table S2**. The estimated variance components from various software tools on simulated dataset.

**Supplementary Table S3**. Comparisons of computational cost on time and memory using different algorithms in HIBLUP to solve the mixed model equation for SSGBLUP model.

**Supplementary File S1**. The running scripts, log information, and recorded computational cost on time and memory for various software tools.

**Supplementary File S2**. The log information and recorded computational cost on time and memory of GCTA and HIBLUP using the simulated UK Biobank scale dataset.
