# Supplementary material for: HIBLUP: an integration of statistical models on the BLUP framework for efficient genetic evaluation using big genomic data
Source: Nucleic Acids Res. 2023 Feb 22;51(8):3501–12. doi: 10.1093/nar/gkad074 (PMC10164590; doi:10.1093/nar/gkad074)
Supplement: gkad074_Supplemental_Files [file gkad074_supplemental_files.zip › Supplementary Notes.docx]

**Supplementary Notes**

***Construction of H and its inverse matrix in SSGBLUP model***

The realized relationship matrix (H) in SSGBLUP model can be expressed as (1,2):

$$\boldsymbol{H=}\left[ \begin{matrix} \boldsymbol{A}_{\boldsymbol{11}}\boldsymbol{+}\boldsymbol{A}_{\boldsymbol{12}}\boldsymbol{A}_{\boldsymbol{22}}^{\boldsymbol{-1}}\left( \hat{\boldsymbol{G}}\boldsymbol{-}\boldsymbol{A}_{\boldsymbol{22}} \right)\boldsymbol{A}_{\boldsymbol{22}}^{\boldsymbol{-1}}\boldsymbol{A}_{\boldsymbol{12}}^{\boldsymbol{T}} & \boldsymbol{A}_{\boldsymbol{12}}\boldsymbol{A}_{\boldsymbol{22}}^{\boldsymbol{-1}}\hat{\boldsymbol{G}} \\ \hat{\boldsymbol{G}}\boldsymbol{A}_{\boldsymbol{22}}^{\boldsymbol{-1}}\boldsymbol{A}_{\boldsymbol{12}}^{\boldsymbol{T}} & \hat{\boldsymbol{G}} \end{matrix} \right]$$

where the matrices $\boldsymbol{A}_{\boldsymbol{11}}$, $\boldsymbol{A}_{\boldsymbol{22}}$**,** and $\boldsymbol{A}_{\boldsymbol{12}}$ are submatrices of pedigree-based additive genetic relationship matrix $\boldsymbol{A}$ that contain relationships among non-genotyped, among genotyped, and between non-genotyped and genotyped individuals, respectively. $\hat{\boldsymbol{G}}$ is the adjusted matrix from the genomic relationship matrix (GRM) $\boldsymbol{G}$ (3), which can be calculated by various algorithms (4-6). $\boldsymbol{A}_{\boldsymbol{22}}^{\boldsymbol{-1}}$ is the inverse of $\boldsymbol{A}_{\boldsymbol{22}}$, which can be computed using the method described by Colleau (7) or by a sparse solver $\boldsymbol{A}_{\boldsymbol{22}}^{\boldsymbol{-1}}\boldsymbol{=}\boldsymbol{A}^{\boldsymbol{22}}\boldsymbol{-}\boldsymbol{A}^{\boldsymbol{21}}{\boldsymbol{(}\boldsymbol{A}^{\boldsymbol{11}}\boldsymbol{)}}^{\boldsymbol{-1}}\boldsymbol{A}^{\boldsymbol{12}}$ with $\boldsymbol{A}^{\boldsymbol{ii}}$ being the partition of $\boldsymbol{A}^{\boldsymbol{-1}}$. And the inverse of H matrix that is required for MME-based algorithm can be derived from the following equation:

$$\boldsymbol{H}^{\boldsymbol{-1}}\boldsymbol{=}\boldsymbol{A}^{\boldsymbol{-1}}\boldsymbol{+}\left[ \begin{aligned} \boldsymbol{&0 &0} \\ \boldsymbol{&0 &}\boldsymbol{G}^{\boldsymbol{-1}}\boldsymbol{-}\boldsymbol{A}_{\boldsymbol{22}}^{\boldsymbol{-1}} \end{aligned} \right]$$

where $\boldsymbol{G}^{\boldsymbol{-1}}$ is the inverse of$\boldsymbol{G}$.

***The APY strategy***

The Algorithm for Proven and Young (APY) strategy considers that the genotyped individuals could be divided arbitrarily into core ($\boldsymbol{c}$) and noncore ($\boldsymbol{n}$) groups (8,9), the inverse of GRM can be calculated only on the condition of the genotypic information of core animals as follows:

$$\boldsymbol{G}^{\boldsymbol{-1}}\boldsymbol{=}\left[ \begin{matrix} \boldsymbol{G}_{\boldsymbol{cc}}^{\boldsymbol{-1}} & \boldsymbol{0} \\ \boldsymbol{0} & \boldsymbol{0} \end{matrix} \right]\boldsymbol{+}\left[ \begin{aligned} \boldsymbol{-}\boldsymbol{G}_{\boldsymbol{cc}}^{\boldsymbol{-1}}\boldsymbol{G}_{\boldsymbol{cn}} \\ \boldsymbol{I} \end{aligned} \right]\boldsymbol{M}_{\boldsymbol{nn}}^{\boldsymbol{-1}}\left[ \begin{matrix} \boldsymbol{-}\boldsymbol{G}_{\boldsymbol{nc}}\boldsymbol{G}_{\boldsymbol{cc}}^{\boldsymbol{-1}} & \boldsymbol{I} \end{matrix} \right]$$

where $\boldsymbol{G}_{\boldsymbol{cc}}^{\boldsymbol{-1}}$ is the inverse matrix of the GRM among core groups, $\boldsymbol{G}_{\boldsymbol{nc}}$ is the inverse matrix of the GRM between noncore and core groups, $\boldsymbol{M}_{\boldsymbol{nn}}$ is a diagonal matrix of genomic Mendelian sampling terms, with diagonal elements equal to $\boldsymbol{m}_{\boldsymbol{ii}}\boldsymbol{=}\boldsymbol{g}_{\boldsymbol{ii}}\boldsymbol{-}\boldsymbol{g}_{\boldsymbol{ic}}\boldsymbol{G}_{\boldsymbol{cc}}^{\boldsymbol{-1}}\boldsymbol{g}_{\boldsymbol{ci}}$, where $\boldsymbol{g}_{\boldsymbol{ii}}$ is the $\boldsymbol{i}_{\boldsymbol{th}}$ diagonal element of $\boldsymbol{G}_{\boldsymbol{nn}}$, $\boldsymbol{g}_{\boldsymbol{ic}}$ is the $\boldsymbol{i}_{\boldsymbol{th}}$ row of $\boldsymbol{G}_{\boldsymbol{nc}}$, and $\boldsymbol{I}$ is the identity matrix.

**References**

1. Christensen, O.F. and Lund, M.S. (2010) Genomic prediction when some animals are not genotyped. *Genetics Selection Evolution*, **42**, 1-8.

2. Aguilar, I., Misztal, I., Johnson, D., Legarra, A., Tsuruta, S. and Lawlor, T. (2010) Hot topic: a unified approach to utilize phenotypic, full pedigree, and genomic information for genetic evaluation of Holstein final score. *Journal of dairy science*, **93**, 743-752.

3. Christensen, O., Madsen, P., Nielsen, B., Ostersen, T. and Su, G. (2012) Single-step methods for genomic evaluation in pigs. *animal*, **6**, 1565-1571.

4. VanRaden, P.M. (2008) Efficient methods to compute genomic predictions. *Journal of dairy science*, **91**, 4414-4423.

5. Yang, J., Benyamin, B., McEvoy, B.P., Gordon, S., Henders, A.K., Nyholt, D.R., Madden, P.A., Heath, A.C., Martin, N.G. and Montgomery, G.W. (2010) Common SNPs explain a large proportion of the heritability for human height. *Nature genetics*, **42**, 565-569.

6. Vitezica, Z.G., Legarra, A., Toro, M.A. and Varona, L. (2017) Orthogonal estimates of variances for additive, dominance, and epistatic effects in populations. *Genetics*, **206**, 1297-1307.

7. Colleau, J.-J. (2002) An indirect approach to the extensive calculation of relationship coefficients. *Genetics Selection Evolution*, **34**, 1-13.

8. Misztal, I. (2016) Inexpensive computation of the inverse of the genomic relationship matrix in populations with small effective population size. *Genetics*, **202**, 401-409.

9. Misztal, I., Legarra, A. and Aguilar, I. (2014) Using recursion to compute the inverse of the genomic relationship matrix. *Journal of dairy science*, **97**, 3943-3952.
